# Factors influencing place of delivery for pastoralist women in Kenya: a qualitative study

**DOI:** 10.1186/s12905-016-0333-3

**Published:** 2016-08-09

**Authors:** Tanya Caulfield, Pamela Onyo, Abbey Byrne, John Nduba, Josephat Nyagero, Alison Morgan, Michelle Kermode

**Affiliations:** 1 The Nossal Institute for Global Health, School of Population and Global Health, University of Melbourne, Level 4, Alan Gilbert Building, 161 Barry St, Carlton, Victoria 3010 Australia; 2 Amref Health Africa, Amref Health Africa Headquarters, Langata Road, Wilson Airport, Nairobi, Kenya

**Keywords:** Pastoralist, Traditional birth attendants, Homebirths, Skilled birth attendants, Kenya, Culture

## Abstract

**Background:**

Kenya’s high maternal mortality ratio can be partly explained by the low proportion of women delivering in health facilities attended by skilled birth attendants (SBAs). Many women continue to give birth at home attended by family members or traditional birth attendants (TBAs). This is particularly true for pastoralist women in Laikipia and Samburu counties, Kenya. This paper investigates the socio-demographic factors and cultural beliefs and practices that influence place of delivery for these pastoralist women.

**Methods:**

Qualitative data were collected in five group ranches in Laikipia County and three group ranches in Samburu County. Fifteen in-depth interviews were conducted: seven with SBAs and eight with key informants. Nineteen focus group discussions (FGDs) were conducted: four with TBAs; three with community health workers (CHWs); ten with women who had delivered in the past two years; and two with husbands of women who had delivered in the past two years. Topics discussed included reasons for homebirths, access and referrals to health facilities, and strengths and challenges of TBAs and SBAs. The data were translated, transcribed and inductively and deductively thematically analysed both manually and using NVivo.

**Results:**

Socio-demographic characteristics and cultural practices and beliefs influence pastoralist women’s place of delivery in Laikipia and Samburu counties, Kenya. Pastoralist women continue to deliver at home due to a range of factors including: distance, poor roads, and the difficulty of obtaining and paying for transport; the perception that the treatment and care offered at health facilities is disrespectful and unfriendly; lack of education and awareness regarding the risks of delivering at home; and local cultural values related to women and birthing.

**Conclusions:**

Understanding factors influencing the location of delivery helps to explain why many pastoralist women continue to deliver at home despite health services becoming more accessible. This information can be used to inform policy and program development aimed at increasing the proportion of facility-based deliveries in challenging settings.

## Background

The fifth Millennium Development Goal aimed to reduce the 1990 maternal mortality ratios by three quarters by the end of 2015, and the main strategy for achieving this goal was increasing the proportion of women delivering with skilled birth attendants (SBAs). An SBA is an accredited health professional such as a midwife, doctor or nurse, who has been trained to manage normal pregnancies, childbirth and the immediate postnatal period, and to identify, manage and, if indicated, refer women and newborns experiencing complications [1]. In 2013, the World Health Organization estimated that 289,000 women died during or following pregnancy and delivery [2], and more than half of these deaths occurred in sub-Saharan Africa.

Women in sub-Saharan Africa have the highest rate of maternal mortality in the world, with 510 maternal deaths per 100,000 live births [3], mostly from preventable causes. Many women in sub-Saharan Africa do not receive skilled health care during pregnancy and delivery, with a large proportion delivering at home assisted by traditional birth attendants (TBAs). TBAs are lay persons (mostly women) who assist women during childbirth and initially acquire their skills by delivering babies or through apprenticeship to other TBAs [4, 5]. However, most TBAs are unskilled and unable to manage the complications that occur in at least 15 % of deliveries [6]. A range of factors prevent women in sub-Saharan Africa from obtaining quality health care from the formal sector including living a long distance from health facilities, poor roads, lack of transport, poverty, cultural practices, lack of information, and poor quality health services [7–13].

Kenya has a high maternal mortality ratio of approximately 400 per 100,000, which has remained largely unchanged since 2005 [3]. One explanation for this is the low proportion of women delivering in health facilities attended by SBAs; nationally, less than half (43 %) deliver with an SBA [14]. In an effort to reduce maternal mortality, the Kenyan Government is promoting skilled birth attendance and proscribing births attended by TBAs. They have also made health facility deliveries available free of charge. Also, the Beyond Zero Campaign, launched by Kenya’s First Lady Margaret Kenyatta, aims to eradicate preventable maternal and child mortality in Kenya through the provision of fully equipped mobile clinics to increase women’s access to quality services in all counties. Despite these strategies, many women continue to give birth at home attended by family members or TBAs, and only resort to formal health care services in the event of complications, often when it is too late for effective intervention. This is especially true for pastoralist women living in the rural and remote areas of Kenya [15], where TBAs are respected members of their communities [16].

There has been limited investigation of the factors influencing place of delivery for women from semi-nomadic pastoralist communities in Kenya. The aim of this paper is to directly address this knowledge gap by investigating the socio-demographic factors and cultural beliefs and practices that influence place of delivery for pastoralist women in Laikipia and Samburu counties. An understanding of these determinants is important for the development of relevant strategies, policies and programs to improve uptake of skilled birth attendance. This paper draws on findings from the first qualitative phase of a mixed methods study that was a partnership between: the Nossal Institute for Global Health, University of Melbourne (Melbourne, Australia); Amref Health Africa (Nairobi, Kenya); the Mother’s Union of the Anglican Church, Kenya (MUACK) (Nanyuki, Kenya); and the relevant County Health Ministries. The study was funded by the Australian Government’s Department of Foreign Affairs and Trade (DFAT), and was conducted in the two counties where MUACK is currently implementing a five year, DFAT-funded maternal and child health project.

### Study context

Laikipia and Samburu are semi-arid, sparsely populated counties located in the Rift Valley region of north Kenya. Both counties experience unreliable rainfalls and frequent droughts leading to water shortages and food insecurity. Each county is divided into group ranches, which consist of a number of small villages or homesteads. A group ranch is principally an organisational structure defined as a ‘livestock production system or enterprise where a group of people jointly own freehold title to land, maintain agreed stocking levels and herd their livestock collectively which they own individually’ [17]. Members of each group ranch are connected through kinship and traditional arrangements of land rights. All group ranches in Laikipia and Samburu are remotely located, and access is limited by poor roads, which can be impassable in the wet season.

The majority of pastoralists in Samburu and Laikipia counties were historically nomadic but have now adopted a semi-nomadic lifestyle; even though they have settled in permanent communities, some family members, mainly men, move livestock seasonally for water and pasture. Their livestock are a source of household food, transport and trade, and are essential for survival and sustenance [16]. Women make a major contribution to the pastoralist lifestyle, but do not generally participate in decision-making, including decisions directly affecting their own lives. Pastoralist societies are patriarchal, and pastoralist women have limited access to and control over productive assets including livestock and land. In many patriarchal societies, the health status of women and children is poor, particularly in communities where women’s decision-making abilities and control over money is limited [18]. In the two counties, formal health facilities providing a range of basic services including maternal and child health care are available, but a high proportion of women (92 %) still deliver at home with the assistance of a TBA, a family member or alone [19]. This is much higher than the Kenyan national average of 56 and 66 % for the Rift Valley region [14].

## Methods

Data collection took place in five group ranches in Laikipia County (Chumvi, Naibor, Makurian, Morupusi, and Tiamamut) and three group ranches in Samburu County (Longewan, Kisima, and Kirimon). The project commenced with engagement of stakeholders and permission from community leaders. Semi-structured in-depth interviews and focus group discussions (FGDs) were conducted primarily between October 2013 and March 2014 – three additional interviews were conducted in December 2014. Fifteen interviews were conducted in Kiswahili, Kenya’s national language, by the study’s research officer. The interviews were with seven SBAs (five female and two male) located at local health facilities; and eight key informants (two male and two female Community Development Committee (CDC) members, two district health managers, and two health facility in-charge personnel).

Local research assistants were recruited and trained in qualitative research methods and FGD facilitation. The research assistants conducted a total of 19 FGDs across the eight group ranches. The FGDs were conducted in the local Maa language and involved a range of respondents: four FGDs with TBAs; three with community health workers (CHWs); five with women who delivered in the past 2 years with a TBA; three with women who delivered in the past 2 years with an SBA; two with women who delivered in the past 2 years without a TBA or SBA; and two with husbands of women who delivered in the past 2 years. CHWs are women and men who have received brief intensive training, and are deployed in each group ranch through the Ministry of Health’s ‘Community Strategy’. A component of their role is to encourage women to attend health facilities for antenatal care and delivery.

The interviews and FGDs took approximately 60–90 min to complete, and were audio-recorded, transcribed and translated from Swahili and Maa into English by the local research team. The topic guides (available from the authors on request) were developed taking into account existing literature and the purpose of the study, and were piloted and revised. They covered reasons for home based deliveries and facility based deliveries, access and referrals to facilities, strengths and challenges of TBAs and SBAs, and strategies for improved care and collaboration. Pictures representing pregnancy, birth, birth complications, and the different types of health facilities were created by a local artist and used as prompts to stimulate discussion during the FGDs with TBAs and community women.

Following transcription of all interviews and FGDs, two researchers analysed the data (TC and AB) using a thematic analysis approach [20]. One researcher used NVivo and the other analysed the data manually. The initial analysis adopted a deductive approach using the topic guides to identify themes. A re-reading of the transcripts using an inductive approach identified emerging sub-themes from data; sub-themes were founded on recurring concepts in the data. The researchers met to review analysis outcomes and ensure thematic concordance. Transcripts were coded and thematically categorised. The data analysis steps are summarised in Table 1.

**Table 1:** Themes that emerged from FGDs and semi-structured interviews

Topics	Themes
Accessing health facilities	Remote locations of group ranches
	- long distance to health facilities
	- poor roads
	- limited transport
	- cost of transport
Perceived quality of SBA care	Poor quality services
	- negative attitudes of SBAs
	- verbal and/or physical abuse
	- left alone during delivery
	- cold environment
	Shame
	- being naked during facility deliveries
	- being attended by male doctors or nurses
Education	Intersection of education and gender
	- women’s lack of health awareness
	- men’s ignorance of women’s health
	- men’s control of decision-making
Preference for home births	TBAs are highly valued community members
	- high prestige of TBAs in community
	- community trust and confidence in TBAs
	- ease of availability
	- familiarity to community members
	Observations of traditions
	- birth rituals can be observed
	- cultural practices and beliefs about births are respected
	- association of illness with health facilities
	Weakness vs bravery
	- women who deliver alone are courageous
	- women who deliver in health facilities are weak
	Unassisted births
	- ease of delivering alone
	- able to conceal non-circumcision from others
	Working throughout pregnancy
	- delivery date unknown
	- short or no labour pain
	- women’s work responsibilities

Following final analysis, interim findings were presented to CDC members from all group ranches to ensure that they accurately captured and reflected the experiences of their communities; all CDC members were supportive of the findings presented.

Ethics approval was obtained from the Ethics and Scientific Review Committee (ESRC) of AMREF (Kenya) and the Human Research Ethics Committee (HREC) at the University of Melbourne (Australia). All study respondents were provided information regarding the study prior to all FGDs and interviews and verbal consent was obtained. Respondents were provided with a small payment in recognition of their time (~USD 4).

## Results and discussion

In this section we present the findings and discuss how socio-demographic factors and cultural beliefs and practices determine where pastoralist women deliver. The findings are organised according to the themes and sub-themes identified through the process of data analysis, which included access to health facilities, quality of care in health facilities, education, and women’s preferences for homebirths. Certain sub-themes are closely interrelated, making it difficult to examine each one separately. For this reason, sub-themes such as distance, roads, and transport issues are discussed together under the broader theme of accessing health facilities, while other sub-themes are discussed separately. Additionally, the implications of the findings for women’s access to health facilities for safer delivery are discussed.

### Accessing health facilities: long distances, poor roads and limited transport

In both Laikipia and Samburu counties, the majority of women and men said that distance to the health facilities contributed to the continuing practice of home births. Although most communities were no longer fully nomadic, the group ranches are remotely located, making delivery in health facilities challenging. Unable to walk the long distance to a health clinic whilst pregnant, the majority of women delivered at home with a TBA, female family member or neighbour, and some delivered alone. Women who delivered with a TBA explained how distance deters women from delivering in health facilities.

When you feel labour pains, you just call [a TBA] to the home. … The hospital is far and [there is] no maternity nearby, so you decide to deliver at home. (Kirimon, FGD with women delivered by TBA)

Distance to the health facility is a big problem for women in some villages. The roads are not passable during rainy seasons so many women will not think of going to the hospital. (Longewan, FGD with women delivered by TBA)

Many of our people live far away; it is the kilometres that bring the problems. You know when one starts to feel the pains, when she begins to walk, she can deliver in the forest or at home. (Morupusi, Interview with female CDC member)

Adding to the problem of distance was the limited availability of transport on the group ranches. To travel to health facilities, families have to hire a local vehicle, which may or may not be available when needed, and during the rainy season some roads become impassable. Although the majority of study respondents knew that giving birth in a health facility was free, the cost of transport was prohibitive for most pastoralist families. The cost of hiring a local car or motorbike ranged from KSH1500 up to KSH4000 (≈USD 16–42), depending on the distance between the group ranch and the facility. The economic poverty of pastoralists contributed to their low uptake of maternal health services.

Now people can … take their women to hospitals, people have seen the importance of going for a hospital delivery. It’s only the transport that is a challenge. This cost is high for many people. Many things go hand in hand, for example during the rainy season, the transport cost is high and sometimes no car can travel on the road. (Longewan, FGD with husbands)

Some walk but those who can’t walk, they hire a vehicle which sometimes cost KSH3000 or more depending on the distance. When you go to Maralal it’s very expensive, a whole cow will have to be sold. (Longewan, FGD with TBAs)

The hospital is far and sometimes the husband doesn’t have money to take the woman to the hospital. The furthest village is 7 km and we have to pay KSH4000 to hire the car. (Kirimon, FGD with women delivered by TBA)

We have to look for a vehicle to hire. Sometimes we don’t find the cars and in this case we make the woman walk. We have to first look for network coverage then we call the owner of the car and negotiate the cost of transport. It costs approximately KSH6000 one way … It takes two to three hours to get to the facility depending on the distance from the village and the roads are not good either. (Longewan, FGD with women delivered by TBA)

To avoid incurring these costs, some women who live relatively close to a health facility walk two or more hours to the facility to give birth. For women who are in the late stages of pregnancy or in labour, the walk to a health facility is slow and difficult.

Some go by foot slowly, − slowly until they get there, while others use a car at a cost of KSH2000. By foot it takes over two hours, by car it is 15 minutes. (Kisima, FGD with women delivered by SBAs)

If women need to go to hospital but they don’t have the money for a motorbike, they will walk very slowly to the dispensary. (Makurian, FGD with women not delivered by TBA or SBA)

Other researchers have similarly identified that distance, poor roads, and cost and lack of transport influence decisions about place of delivery [8, 13, 21–25]. Similarly, findings from the Kenyan Demographic and Health Survey [14] indicates that one of the main reasons for Kenyan women not delivering in a health facility was distance and absence of transport (cited by 42 % of those who delivered at home), which are two of the three delays identified in the classic Thaddeus and Maine study [24]. Distance and lack of transport options are both barriers to reaching health facilities as well as a disincentive to seeking health care [24]. The barrier of distance contributes to the cultural kudos of TBAs, who are nearly always available to assist women during delivery, something that is highly valued by communities [13]. Improving ambulance services in remote areas can help to increase the number of women delivering in facilities [26]. Through an initiative in Ethiopia, a government primary health care unit provided an ambulance service for remote rural areas to bring pregnant women to the health centre for delivery. While this increased women’s transportation options to the health facility, the initiative encountered a number of challenges, limiting its success. These included: lack of fuel provision for the ambulance; smaller health posts were not included on the ambulance route; insufficient number of ambulances to service community need; and impassability of roads [26]. The success of this strategy in the case of pastoralist women would be contingent on overcoming similar challenges, such as ensuring roads are passable and fuel supplies are adequate.

### Perceived quality of care in health facilities

Many community respondents said that they were deterred from going to health facilities due to the negative attitudes and low quality services provided by some SBAs. Some women said they preferred home births because they had heard about or had directly experienced SBAs being verbally or physically abusive to pastoralist women in health facilities. These perceptions and experiences reinforced continued adherence to traditional birthing practices, which were viewed more positively.

I just deliver at home so I can get someone to hold me. In hospital you are beaten, at home you get someone who comforts you till you deliver … At home you get many women attending you. (Tiamamut, FGD with TBAs)

The women say they are beaten by the nurses. They are not attended well by the nurses. (Chumvi, FGD with husbands)

Doctors keep a distance and keep watching you … During labour pains you meet a doctor who slaps you seriously. At home you say let me stay where nobody beats me up. (Longewan, FGD with TBAs)

Reports that women are frequently left alone during facility-based deliveries also deterred women from attending health facilities. It is customary for pastoralist women to be held by other women during delivery. Having many women present during the birth provided support and comfort and was preferable to what was perceived to be the uncaring attitude of SBAs.

The other thing why we don’t like [SBAs] is because when you are in labour pain they don’t have time with you, you can’t even find one [SBA] to hold you. At home if you call someone they will hurry and hold you till you give birth. (Naibor, FGD with women delivered by SBAs)

In hospital during labour pains, you can faint and nobody will console you but at home the women will hold you. (Morupusi, FGD with women delivered by TBAs)

At home, you can get someone to hold you. In hospital you are beaten, at home you get someone who comforts you until you deliver … At home you get many women attending to you. (Tiamamut, FGD with TBAs)

Most women talked about the health facility as an unpleasant place to deliver. They said it was a cold environment, and this was another reason for many women delivering at home, where the environment is intentionally warmed at the time of delivery. Community members believed that a warm house ensured a speedy delivery and protected mother and child from illness.

We believe that a delivering woman must be in a warm place and it’s cold in the dispensary … we protect the mother and the child from cold. (Longewan, FGD with women delivered by TBAs)

In our culture, we believe that you will deliver quickly when you are in a warm place so that’s why many deliver at home. (Chumvi, FGD with women delivered by TBAs)

After delivery, the cold affects the woman and child. We give them some herbs and make sure the house is always warm. (Makurian, FGD with TBAs)

These findings are not unique as other studies have identified poor relationships between women and health care providers as one of the major barriers to the uptake of formal maternal care by women in Kenya and other developing countries [23, 25–27]. In Kenya, for example, Izugbara et al. [27] argue that there is a mismatch between the values, needs, and sensitivities of women and health service providers, which hinders formal maternal health services; Kenyan health care providers are often unfriendly and dismissive of women, particularly poor women. Bedford et al. [8] argue that basic features of maternal services in rural Ethiopia, such as allowing relatives and/or neighbours to support women during deliveries, allowing women to choose preferred delivery positions, and ensuring health facility environments are culturally responsive to the needs of women are simple measures that can be adopted to make services socially acceptable to women.

#### Shame

The risk of being shamed was identified by several women as a reason for not attending health facilities. Women said they preferred to deliver at home as facility-based deliveries required women to be naked, which was considered to be shameful. During homebirths, TBAs cover women with a blanket or sheet so that women’s bodies cannot be seen.

If you deliver at the hospital, they see it as shame. Because they say all your clothes are removed … you will stay without any clothes and you deliver naked. (Longewan, Interview with female Community Development Committee [CDC] member)

Women don’t want to deliver at the hospital because they don’t want to be stitched and they are also told to remove their clothes; women are covered at home. (Longewan, FGD with TBAs)

The shame of being naked was exacerbated if the nurse or doctor was male. Some women stated that if women went to the dispensary and found a male SBA there, they would return home without treatment.

[In our] culture, some don’t go to hospital because they don’t want to be helped by male doctors; women don’t want them to see their private parts. (Naibor, FGD with CHWs)

At home we are helped by TBAs and other women. You go to the dispensary and you find a male nurse there so you go back home. (Longewan, FGD with women delivered by TBAs)

In our culture, a male is not allowed to take care of the mother during the delivery period. (Kirimon, Interview with male CDC member)

Recent studies from Ethiopia highlight how factors such as disrespect for women’s modesty, uncomfortable health facilities, and separation from family members during delivery dissuade women from seeking care at health facilities [23, 26]. One study in Ethiopia also found that women experience a sense of shame associated with having men examining or delivering them [26]. Deploying mainly female staff to complement male doctors and nurses in health facilities serving pastoralist women may encourage better attendance during pregnancy and delivery. Investing in patient-friendly approaches to delivery can ameliorate the more formal aspects of maternal care [26]. Sensitising health providers regarding the need to understand and respect community beliefs and practices is likely to increase the uptake of facility-based services [28]. Services that consider women’s modesty, comfort, and delivery preferences are necessary if pastoralist women’s access to facility-based deliveries in Laikipia and Samburu counties is to be increased.

### Education

Study respondents in both Laikipia and Samburu suggested that lack of education was a reason for women not having facility-based deliveries. Although awareness of the importance of facility-based deliveries was being promoted by the CHWs and others, many respondents maintained that only better educated or younger women delivered with SBAs. Community members suggested:

Things are changing. The youth prefer hospitals but old people don’t like hospitals. (Tiamamut, FGD with women delivered by TBAs)

If your husband has money or if women are educated, they like going to the big hospital. (Tiamamut, FGD with TBAs)

Education is encouraging women to go to hospital. Learned families go to the hospital for delivery. (Longewan, FGD with husbands)

Educational opportunities for pastoralist men in Laikipia and Samburu are also limited. Some women identified men’s ignorance of women’s health during pregnancy as a potential obstacle to women receiving formal health care, as husbands usually decide where the women should seek care.

No care is given unless she is sick. She will go to fetch firewood alone, she will do all the work alone … But men are different some will just leave you [to] suffer alone without any help. (Kisima, FGD with women delivered by SBAs)

Women don’t refuse to go to hospital; it is their husbands who don’t want them to go. (Tiamamut, FGD with women delivered by TBAs)

Husbands don’t care about their women and they don’t see them as having any real problem during their pregnancy. (Longewan, FGD with husbands)

When a woman is unwell during her pregnancy, the TBAs or women from the group ranch prescribe treatment, which may include advising the husband to slaughter an animal for blood and meat. The role of men in these situations is significant given that livestock production is the main source of income and prestige for pastoralists; so selling livestock to raise money for health care or killing an animal to give blood or meat to an unwell, pregnant woman is a difficult decision for men.

The husband feels that to give his sheep or bull to just sell for the wife to get something good to take care of her body … he sees that one as a loss. (Longewan, Interview with female CDC member)

If a woman has a problem during pregnancy, women know the herbs to be given; they also inform the husband. For bleeding, we have to slaughter a goat but first the women must inform us before we give permission to slaughter. (Longewan, FGD with husbands)

When a woman is sick, the TBAs give herbs mixed with blood, soup, or milk. The TBAs inform the husband about the sickness and advise him to slaughter [a goat]. This is where the husband plays a big decision making role. (Longewan, FGD with women delivered by TBA)

Education plays an important role by equipping both women and men to make informed decisions about pregnancy and delivery, and can have a significant impact on maternal mortality, even in countries where women have weaker roles in decision-making [29]. Educating men about the benefits of women delivering at a health facility is essential given their role as providers and decision-makers. Activities that promote joint decision-making between women and their husbands have been shown to increase the number of women delivering with SBAs [30, 31].

The remoteness of group ranches also contributes to the widespread lack of educational opportunities for people living in these communities. It was only recently that government and non-government organisations implemented programmes to provide education and literacy classes for children and adults in both counties. Studies have shown that education levels are an important determinant of women using maternal and child health services, and women with higher levels of education are more likely to access them than women without education [12, 28, 32, 33]. Younger women are also more likely to deliver in health facilities compared to older women [34], perhaps because they are better educated. Improving educational opportunities for women, particularly in rural areas, increases the likelihood that they will use available maternal and child health services [11, 35]. Research suggests that there is a strong association with women’s education and SBA utilisation, and in Africa this is significant even at primary levels [36]. Short-term health information programmes can also play an important role in influencing the decisions of women with little or no education about place of delivery [11, 25]. In Laikipia and Samburu counties, CHWs provide specific health related knowledge, including the importance of facility-based deliveries with SBAs, although the impact of this initiative is still to be evaluated.

### Women’s preferences for homebirths

While some of the themes identified above actively deterred women from attending a health facility at the time of pregnancy and delivery, a number of themes emerged that reinforced women’s preference to deliver at home.

#### TBAs are highly valued community members

In all group ranches, home deliveries were the norm; very few women had delivered at a health facility assisted by an SBA. Among the women present during home deliveries were TBAs who assisted women before, during and after birth. TBAs occupy a prestigious place in pastoralist communities because of the health care they provide for women at the time of pregnancy and delivery. Community respondents explained that TBAs confirmed a woman’s pregnancy, gave advice on appropriate diets and workloads, and administered herbs for particular conditions. At the first sign of labour, the women of the *manyatta* (household) send for the TBA. One woman explained that many women:

… want to deliver at home because they see it is a TBA who will deliver them well – she is the one who will light a good fire for them, she will clean them well, so you find that … the majority like to deliver [at home]. (Longewan, Interview with female CDC member)

TBAs help you. You know, we are far from the hospital but when those women are there, they can help us even if we have to go to the hospital. They help us to deliver so I see that this is their goodness. (Morupusi, Interview with female CDC member)

TBAs are available throughout. They encourage you to eat and breastfeed the baby. They are not rude in talking to the mother. (Tiamamut, FGD with women not delivered by TBA or SBA)

TBAs were part of the community and their familiarity and availability meant that women readily relied on them for advice and assistance during pregnancy and birth. A woman could tell a TBA:

… her problems because she is a woman from the family and she understands her; she does not fear the TBA, so she gives her the secret of her body. We see that as the TBA helping the woman in the community a lot. (Longewan, Interview with female CDC member)

TBAs do a great job because these women who are not near the dispensary … must stay close to a TBA; this is a tradition and it is the TBA who looks after her; it is the TBA who checks on the women. (Makurian, Interview with male CDC member)

As other studies from Uganda, Kenya and Nigeria have shown, women’s preference for the services of TBAs contributes to the perpetuation of home births [9, 13, 27, 33]. Izugbara et al.’s [27] study on TBA practices in urban Kenya suggests that women in communities develop trust and confidence in TBAs, because they are perceived as being highly committed to the wellbeing of women and sensitive to their socio-economic and cultural preferences. Women value TBAs for their comprehensive and consistent care during pregnancy and birth, while also understanding and facilitating cultural practices [9]. Despite the trusted and respectful relationships TBAs develop with women during pregnancy and delivery, most TBAs have not been formally trained in maternal and newborn care and are unable to assist women when complications arise. Given the rapport between women and TBAs and the distance between group ranches and health facilities, models that foster collaboration between TBAs and SBAs are likely to improve maternal health outcomes for pastoralist women in Laikipia and Samburu. Other studies suggest that TBAs can successfully escort women to health facilities as part of a referral process [9, 13, 37, 38]. The integration of TBAs with formal health systems has the potential to increase women’s access to skilled birth attendance [25, 39].

#### Observation of traditions

The preference for home births was explained as ‘*a custom*’ or as ‘*it has always been*’. Some CHWs said:

It is our culture. We are following what our ancestors were doing … they delivered several times at home without any problem. (Tiamamut, FGD with CHWs)

It’s our culture. Our parents tell us I gave birth to you in this house so we follow what they did. (Makurian, FGD with women not delivered by TBA or SBA)

There was almost full agreement among the study respondents that tradition was an important factor influencing the popularity of homebirths, with women’s husbands stating:

Homes are warm, it’s our tradition, and [women] are used to it, even this old man was born in these houses. (Chumvi, FGD with husbands)

It’s been our tradition from the beginning. Maasai women are ignorant. They may have labour pain but she can’t tell anyone; when she is about to deliver, that is the time she calls people. (Tiamamut, FGD with women delivered by TBAs)

Many respondents identified specific cultural practices and beliefs that influenced the place of delivery. Some said that a customary announcement is made when a baby is born and that this cannot be done at the hospital. Others suggested that having family members help with the delivery ensures a baby is delivered easily, while some identified superstitious beliefs, such as ensuring blood loss during delivery is kept within the homestead to protect against bewitchment, as the reason for the popularity of homebirths.

Women associated health facilities with illness and would only go if they had complications during labour; and some people had great faith in the traditional herbs used by TBAs to help women experiencing problems during labour. Other authors have identified the concepts of custom or habit as deterrents to health facility deliveries [8, 13]. In rural Ethiopia, custom precludes women from having a facility-based delivery, although they do seek medical intervention at other times [8]. Rural Ethiopian women view health facilities as a place of illness, and believe that ‘normal’ births do not require ‘treatment’, so there is no need to attend health facilities for delivery. Prior experiences shape current expectations so women who deliver without complications are more likely to deliver in the same location again [13].

#### Unassisted births

In both Laikipia and Samburu counties, there were women who had delivered by themselves, which was often related to cultural beliefs about birth. Some believed that if a woman gave birth in the presence of others, the onlookers would have ‘*bad eyes*’ and the baby might die; others said that babies would be ‘*bewitched*’ or ‘*others may take your blood and bewitch you*’ (Naibor, FGD with Women with SBAs). Many also spoke about the ease with which women can deliver if alone.

[There is] a cultural belief … if other people are there the child will not come out easily. (Chumvi, FGD with women delivered by SBAs)

Some believe that if there are many people in the house the baby will not come quickly. (Kisima, FGD with women delivered by TBAs)

Some women want to be alone because when there are many people the child won’t come out. (Tiamamut, FGD with women delivered by TBAs)

The practice of unassisted births is common in other parts of sub-Saharan Africa, such as Uganda and Tanzania [9, 40]. In Uganda, Kwagala [9] found that unassisted births are admired as a cultural ideal, and the labouring women are responsible for achieving a live birth. The pressure to uphold this tradition can lead to negative outcomes for women and their infants, particularly in the case of women with a history of complicated pregnancies and births. Kwagala [9] argues that women and communities need to be educated regarding the importance of mothers’ and children’s wellbeing to counter beliefs that endanger their lives.

In order to uphold tradition, many pastoralist women are circumcised at puberty as a rite of passage to womanhood. Circumcision demonstrates a woman’s maturity and enables her to legitimately participate in society [15]. Circumcision is a guarantor of virginity, purity, chastity, family honour and marks a girl’s readiness for marriage and childbearing [41]. A woman who is not circumcised will always be regarded as a child. Practices such as circumcision are not only central to a woman’s identity in pastoralist communities but influence their health outcomes also. The stigma of being uncircumcised was identified as a theme that influenced where women give birth.

In our culture, Maasai, if a woman is not circumcised, she will be circumcised during her delivery. So to avoid this she might deliver alone. (Naibor, FGD with CHWs)

Interestingly, being uncircumcised was also given as a reason for why women delivered at a health facility; women did not want others in the community to know they were uncircumcised because it could result in shame and marginalisation.

Some women are not circumcised and they don’t want the community to know so they hide their secret. Most of those who come to hospital are not circumcised. (Kisima, FGD with CHWs)

#### Weakness vs bravery

Pastoralist women who deliver alone were described as brave or courageous by some respondents. Concepts of courage and strength were discussed by women in both Laikipia and Samburu, and some women who birthed alone ‘*considered themselves brave and don’t want anybody close to them when they give birth*’ (Kisima, FGD with women delivered by SBAs). Some women perceived this to be standard practice.

There are [women who deliver alone]. They are courageous, they will stay without telling anyone and they give birth when they are alone. (Chumvi, FGD with women delivered by SBAs)

Women sometimes don’t know the time to give birth and they are caught unaware. These women are not cowards. We have those [women] who are cowards and they call the TBA immediately. (Kirimon, FGD with women delivered by TBAs)

Other studies in sub-Saharan Africa have found that women can be perceived as weak if they seek care at health facilities. In a study on birthing choices in eastern Uganda, Kwagala [9] explains that women from the Sabiny community believe that unassisted births are a test of endurance and a marker of being a ‘real woman’. These women are expected to be resilient and show no signs of pain during delivery, otherwise their identity as strong women will be compromised.

#### Working through pregnancy

Some women gave birth alone while engaging in their work. In pastoralist communities, women often do not know the likely delivery date and continue to undertake strenuous work throughout their pregnancy, often at the recommendation of TBAs. Study participants said that some women went into labour whilst away from the *manyatta* attending to work, so delivered alone. Some women experienced short or no labour pains, which made them deliver quickly.

Some deliver alone when they go to graze the animals … some have very short labour pains, which make them deliver sometimes when they are alone. (Makurian, FGD with CHWs)

We are used to delivering at home and it is only complications that we take to hospitals. You can deliver while looking after livestock. For me I delivered while coming from shopping. … Unless the child comes before you reach hospital you just deliver. (Makurian, FGD with TBAs)

There are some [women who] deliver when they are far from home when they go to fetch firewood. (Naibor, FGD with women delivered by SBAs)

Women from poor socioeconomic backgrounds are more likely to work during pregnancy in order to provide food and water for their families; and poverty exposes women to heavy workloads during pregnancy, which potentially contributes to adverse maternal health outcomes [42]. Increasing women’s attendance at ante-natal clinics will inform them of the risk of heavy workloads and enable better birth preparedness, including arranging access to a SBA. Information, education and communication interventions directed towards behaviour change need to address the sociocultural beliefs that perpetuate the practice of unassisted births in remote rural locations [23].

### Limitations

This study has some limitations. Firstly, the findings are based on qualitative data only, so they cannot be generalised. Secondly, the processes of translation and transcription into English may have resulted in original meanings being distorted or a less nuanced understanding of people’s perceptions of the reasons why women deliver at home. Efforts were made to reduce this loss by using two local research assistants who double-checked all transcriptions against audio recordings of discussions to ensure accuracy. Thirdly, it is possible that the socio-demographic and cultural practices and beliefs identified as factors influencing pastoralist women’s place of delivery are context specific, thereby limiting the transferability of the findings.

## Conclusion

This study indicates that socio-demographic characteristics and cultural practices and beliefs substantially influence pastoralist women’s place of delivery in Laikipia and Samburu counties, Kenya. Pastoralist women continue to deliver at home due to a range of factors including: distance, poor roads, and the difficulty of obtaining and paying for transport; the perception that the treatment and care offered at health facilities is disrespectful and unfriendly, which contrasts with the perception of the warm and caring birthing experience provided by TBAs; lack of education and awareness regarding the risks of delivering at home; and local cultural values related to women and birthing. Understanding the factors influencing decisions about the location of delivery helps to explain why many pastoralist women continue to deliver at home, and this information can be used to inform the development of policies and programs aimed at increasing the proportion of facility-based deliveries in this challenging setting, thereby improving the maternal and newborn health of pastoralist communities.
